# Cholecystitis Associated With a Suprahepatic Gallbladder: Surgical Challenges and Technical Considerations

**DOI:** 10.7759/cureus.108960

**Published:** 2026-05-16

**Authors:** Tomoko Takagishi, Hirofumi Ishikawa, Kiyoshi Endo, Koji Enomoto

**Affiliations:** 1 Department of Surgery, Ikoma City Hospital, Ikoma, JPN; 2 Department of Surgery, Saiseikai Gose Hospital, Gose, JPN

**Keywords:** anomalous gallbladder position, cystic duct anomaly, difficult laparoscopic cholecystectomy, laparoscopic cholecystectomy, suprahepatic gallbladder

## Abstract

Suprahepatic gallbladder is a rare anatomical variant in which the gallbladder body and fundus are displaced superiorly along the common hepatic duct, often associated with hypoplasia of the medial segment of the left hepatic lobe. Surgical treatment of cholecystitis in such cases presents significant technical challenges due to the aberrant position of the gallbladder and its proximity to the major bile ducts. The management of two cases of cholecystitis associated with suprahepatic gallbladder is reported.

Case 1 was an 87-year-old woman with cholecystitis and choledocholithiasis. Magnetic resonance cholangiopancreatography (MRCP) identified a suprahepatic gallbladder. Mild dilation of the common bile duct (CBD) was noted, with the presence of endoluminal biliary sludge. In this case, an endoscopic retrograde gallbladder drainage (ERGBD) stent was placed in the cystic duct and an endoscopic retrograde biliary drainage (ERBD) stent was placed in the CBD.

Case 2 was an 85-year-old male with a history of liver cirrhosis and esophageal varices who visited a clinic with gallstone impaction. Imaging revealed Chilaiditi syndrome, featuring a suprahepatic gallbladder positioned anterior to the liver. The fundus of the gallbladder extended superiorly to the level of the diaphragm. Laparoscopic cholecystectomy was completed without bile duct injury or bile duct leakage, but the patient later developed portal and mesenteric venous thrombosis on postoperative day 10, leading to fatal multi-organ failure.

In the management of cholecystitis of suprahepatic gallbladder, we successfully carried out different approaches; one was by ERGBD and the other by laparoscopic cholecystectomy without CBD injury.

## Introduction

Anomalous positioning of the gallbladder is an uncommon but clinically significant finding that can markedly complicate laparoscopic cholecystectomy. Suprahepatic gallbladder is a rare variant in which the gallbladder body and fundus are displaced superiorly along the common hepatic duct, typically in association with hypoplasia of the medial hepatic segment. This anatomical configuration places the gallbladder near major bile duct structures, substantially increasing the risk of bile duct injury during laparoscopic dissection [1]. Several cases of laparoscopic cholecystectomy complicated by Chilaiditi syndrome, defined as the interposition of the large intestine or, sometimes, the small intestine into the subphrenic space, have been described in the literature. The coexistence of Chilaiditi syndrome with suprahepatic gallbladder further compounds surgical difficulty by obscuring the operative field and requiring additional maneuvers for colonic retraction [2]. Herein, we report two cases of cholecystitis in a suprahepatic gallbladder and discuss the technical surgical considerations and pitfalls encountered, with reference to the existing literature.

Anatomy of suprahepatic gallbladder and surgical implications

Suprahepatic gallbladder refers to an anomalous gallbladder position in which the body and fundus are displaced cranially and lie adjacent to or above the liver parenchyma, typically running along the course of the common hepatic duct. This variant is frequently associated with hypoplasia or agenesis of the medial segment, segment IV, of the left hepatic lobe, which is thought to permit the rostral migration of the gallbladder during embryological development [1]. The key surgical implication is that the posterior wall of the gallbladder may directly overlie the common hepatic duct or right hepatic duct, rendering standard dissection of Calot’s triangle hazardous. Preoperative imaging with computed tomography and magnetic resonance cholangiopancreatography (MRCP) is essential for delineating gallbladder anatomy and its relationship to the biliary ductal system [2].

## Case presentation

Case 1

**Figure 1:**
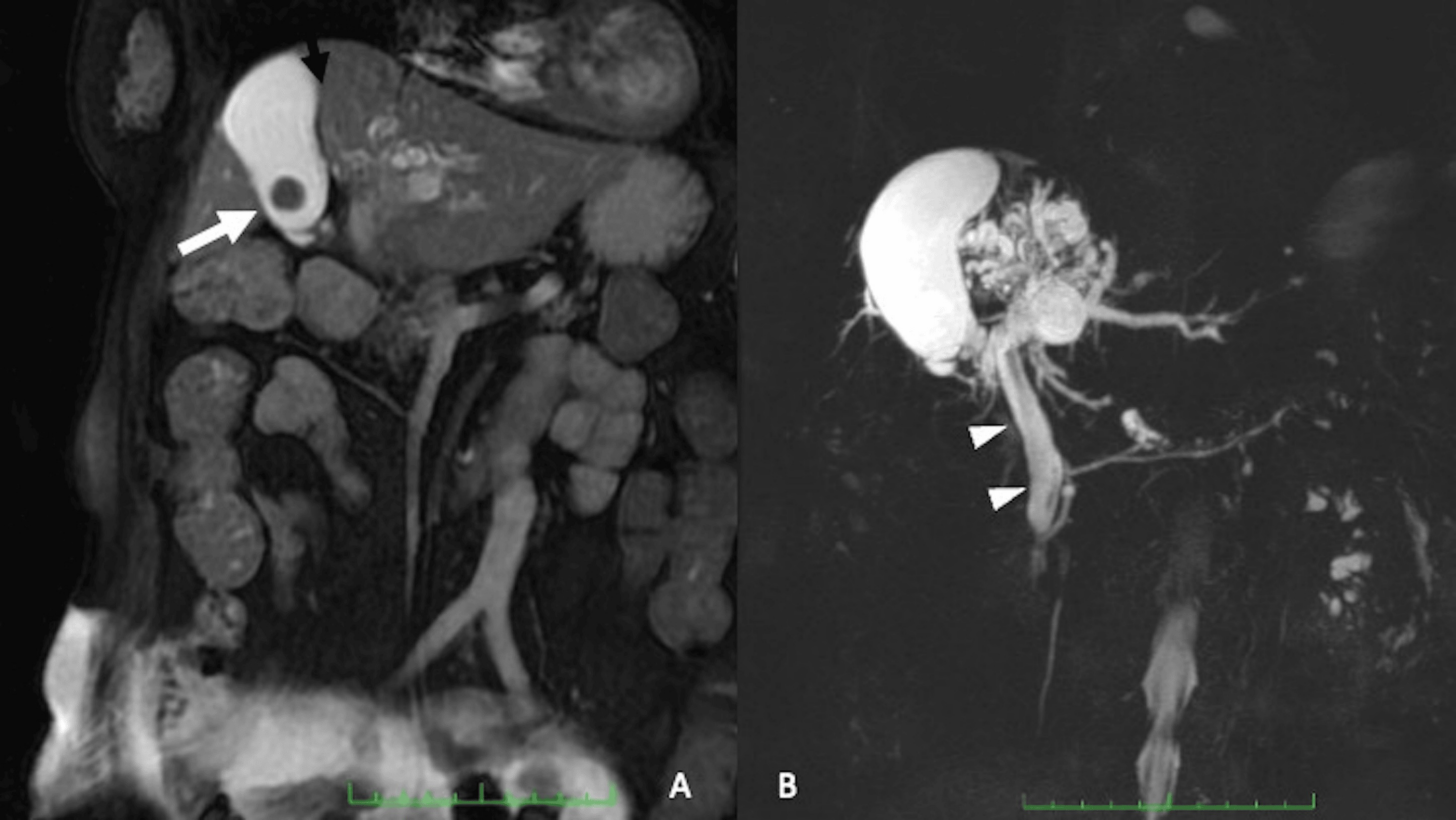
Case 1: Magnetic resonance cholangiopancreatography (MRCP). MRCP showed cholecystitis (A, white arrow) and choledocholithiasis (B, white arrowhead). Sludge-like, friable gallstones were observed in the common bile duct (B, white arrowhead). The gallbladder was positioned between the right and left hepatic lobes, with the cystic duct located deep within the liver parenchyma.

An 87-year-old female with a surgical history of Billroth I reconstruction for gastric cancer presented with fever, malaise, and abdominal pain. MRCP showed cholecystitis and choledocholithiasis, with sludge-like, friable gallstones observed in the common bile duct (CBD; white thin arrow). A suprahepatic gallbladder was identified, with the gallbladder embedded between the right and left lobes of the liver. The cystic duct ran parallel to the CBD, and the gallbladder fundus was adhered to the diaphragm (Figures 1A-1B). Laboratory tests (Table 1) showed CRP 12.45 mg/dL, WBC 10,900/μL, total bilirubin (T-Bil) 4.0 mg/dL, and gamma-glutamyl transpeptidase (γ-GTP) 598 U/L, suggesting obstructive jaundice, although transaminase levels remained within normal limits.

**Table 1:** Laboratory evaluation for Case 1. The reference ranges are based on commonly accepted adult clinical laboratory standards and may vary depending on institutional and regional laboratory practices.

Parameter	Value	Reference range
WBC count	10,900/mm³	3.0-8.6 × 10³/mm³
Hemoglobin	8.6 g/dL	11.6-14.8 g/dL
Platelet count	18.0 × 10⁴/mm³	15.8-35.8 × 10⁴/mm³
C-reactive protein	12.45 mg/dL	0.00-0.14 mg/dL
Aspartate aminotransferase	17 U/L	13-30 U/L
Alanine aminotransferase	23 U/L	7-23 U/L
Alkaline phosphatase	527 U/L	28-113 U/L
Gamma-glutamyltransferase	598 U/L	9-32 U/L
Total bilirubin	4.0 mg/dL	0.4-1.5 mg/dL
Direct bilirubin	2.6 mg/dL	0.1-0.3 mg/dL

**Figure 2:**
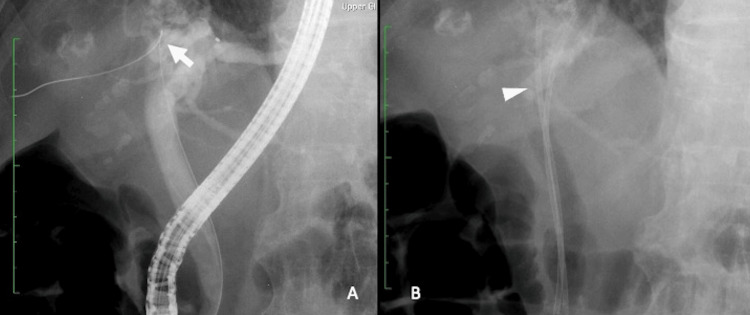
Endoscopic retrograde cholangiopancreatography (ERCP) and endoscopic retrograde biliary drainage (ERBD) treatment. Case 1. Endoscopic retrograde cholangiopancreatography (ERCP) revealed mild dilation of the common bile duct. Although an endoscopic retrograde biliary drainage (ERBD) tube was successfully placed in the common bile duct and a guidewire could be advanced into the gallbladder (A, arrow), the acute angulation of the cystic duct prevented insertion of an endoscopic retrograde gallbladder drainage (ERGBD) tube into the gallbladder; therefore, the ERGBD tube was placed at the cystic duct (B, arrowhead).

Due to the patient’s advanced age and the risk of severe adhesions from the prior gastrectomy, an open approach or laparoscopic cholecystectomy was considered technically prohibitive. Specifically, the gallbladder’s location and the site of the CBD made surgical intervention difficult and high risk. Given the presence of CBD stones and the Billroth I anatomy, endoscopic retrograde cholangiopancreatography (ERCP) was selected as the only viable therapeutic option. ERCP (Figure 2) confirmed a suprahepatic gallbladder and biliary dilation. Although CBD stones were successfully extracted using a basket catheter, an acute angulation between the cystic duct and the neck of the gallbladder prevented gallbladder cannulation with a tube. Consequently, the procedure was completed by placing an endoscopic retrograde gallbladder drainage (ERGBD) stent in the cystic duct and an endoscopic retrograde biliary drainage (ERBD) stent in the CBD.

As post-endoscopic treatment, the patient required one month of hospitalization, including one week of fasting, to achieve resolution of the inflammation. Although she was readmitted two months after discharge for cholangitis, her condition improved following ERBD tube exchange and two weeks of inpatient care. Currently, three years after the initial intervention, the patient remains in good health with no recurrence of cholecystitis.

Case 2

**Figure 3:**
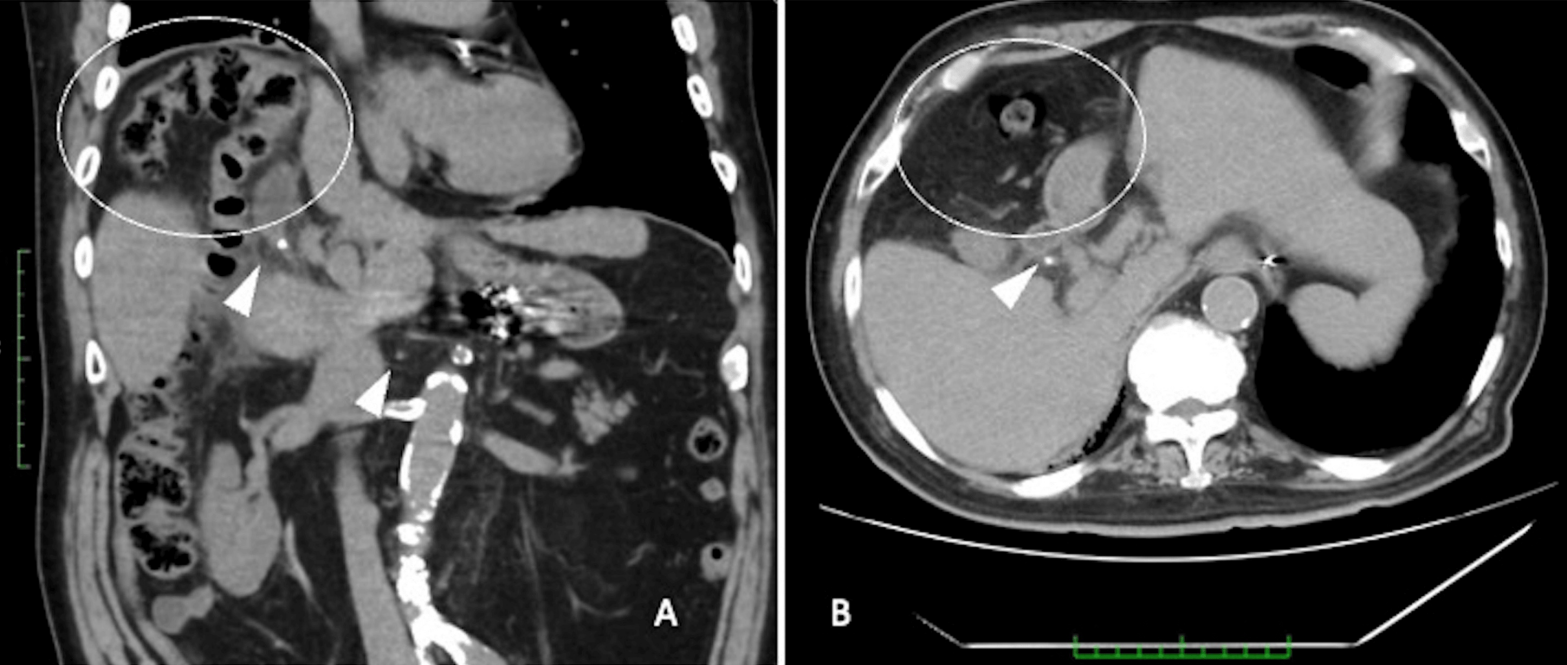
Case 2: Computed tomography (CT). Computed tomography (CT) revealed a gallstone impacted in the cystic duct (A, arrowhead). Additionally, the transverse colon was interposed between the diaphragm and the liver and was in close contact with the gallbladder (A and B, circles).

**Figure 4:**
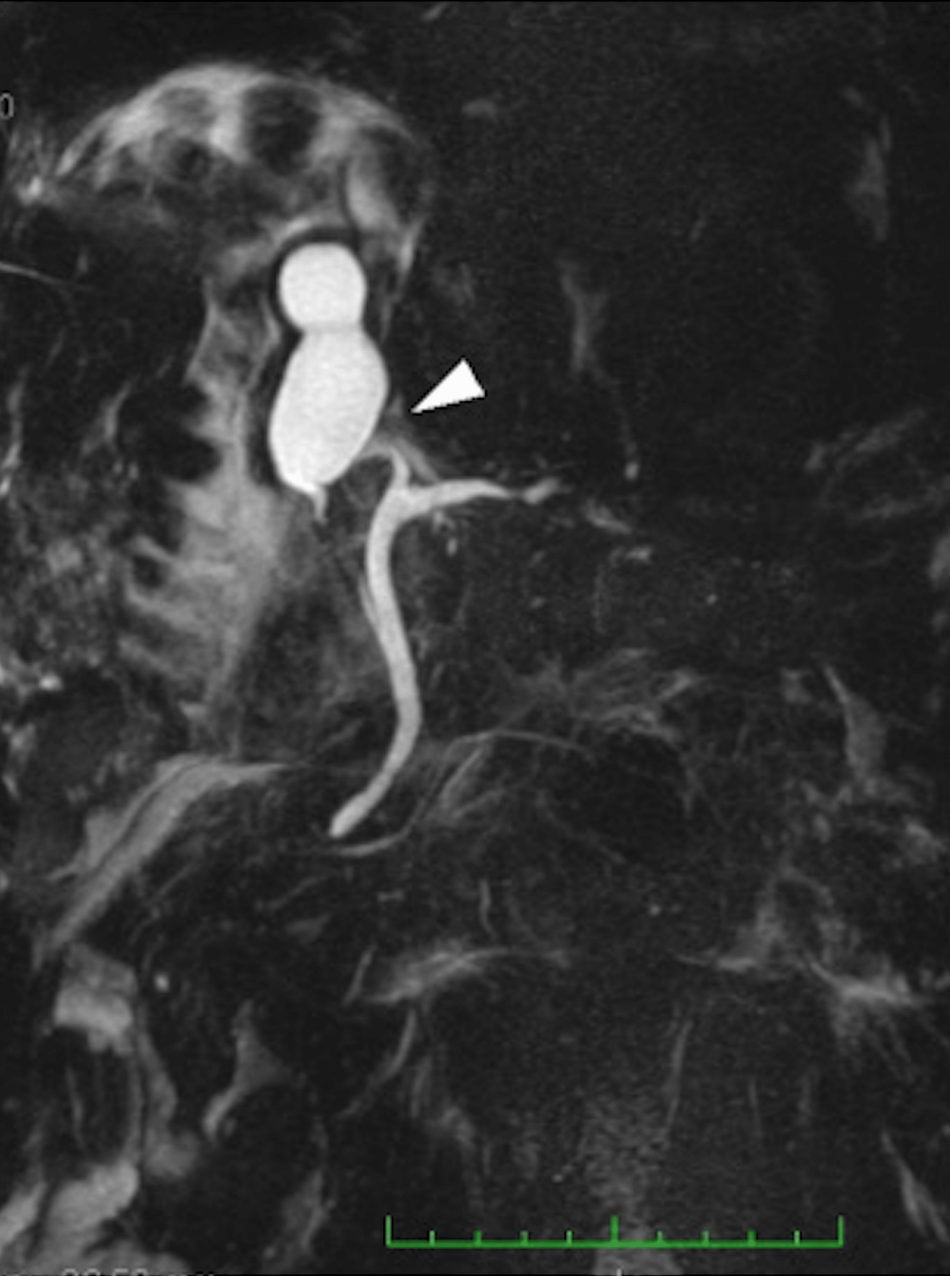
Case 2: Magnetic resonance cholangiopancreatography (MRCP). MRCP revealed atrophy of the right hepatic lobe, with the gallbladder situated along the right intrahepatic bile duct, consistent with a suprahepatic gallbladder (arrowhead).

An 85-year-old male was referred to our hospital with fever and malaise and was diagnosed with cholecystitis. He had a medical history of liver cirrhosis and esophageal varices, the latter of which had been treated with balloon-occluded retrograde transvenous obliteration. CT (Figure 3) revealed hypoplasia of the right hepatic lobe, Chilaiditi syndrome, and a gallstone impacted in the cystic duct, though no stones were identified in the CBD. MRCP revealed atrophy of the right hepatic lobe, with the gallbladder located in front of the liver along the right intrahepatic bile duct, which appeared to be a suprahepatic gallbladder (Figure 4). Laboratory findings (Table 2) indicated CRP 6.02 mg/dL, WBC 11,300/μL, aspartate aminotransferase (AST) 181 U/L, alanine aminotransferase (ALT) 150 U/L, T-Bil 6.4 mg/dL, direct bilirubin (D-Bil) 3.9 mg/dL, and γ-GTP 330 U/L, which indicated liver dysfunction and obstructive jaundice.

**Table 2:** Initial laboratory findings in Case 2.

Parameter	Value	Reference range
WBC count	11,300/mm³	3.0-8.6 × 10³/mm³
Hemoglobin	14.0 g/dL	11.6-14.8 g/dL
Platelet count	8.5 × 10⁴/mm³	15.8-35.8 × 10⁴/mm³
C-reactive protein	6.02 mg/dL	0.00-0.14 mg/dL
Aspartate aminotransferase	181 U/L	13-30 U/L
Alanine aminotransferase	150 U/L	7-23 U/L
Alkaline phosphatase	141 U/L	28-113 U/L
Gamma-glutamyltransferase	330 U/L	9-32 U/L
Total bilirubin	6.4 mg/dL	0.4-1.5 mg/dL
Direct bilirubin	3.9 mg/dL	0.1-0.3 mg/dL

**Figure 5:**
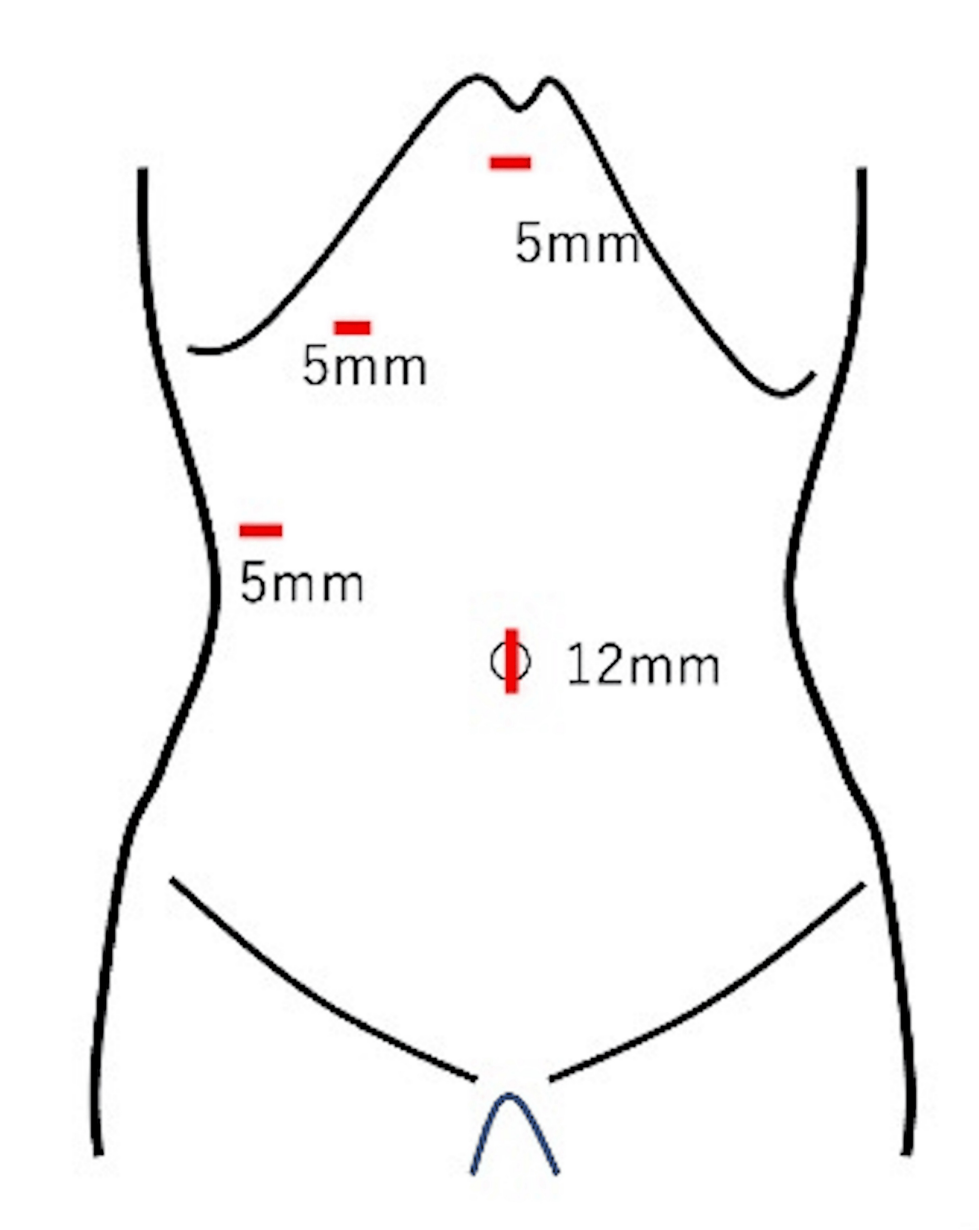
Case 2: Schematic illustration of laparoscopic cholecystectomy. Schematic illustration showing insertion of a 12-mm camera port through the umbilicus and three 5-mm subcostal ports during laparoscopic cholecystectomy.

**Figure 6:**
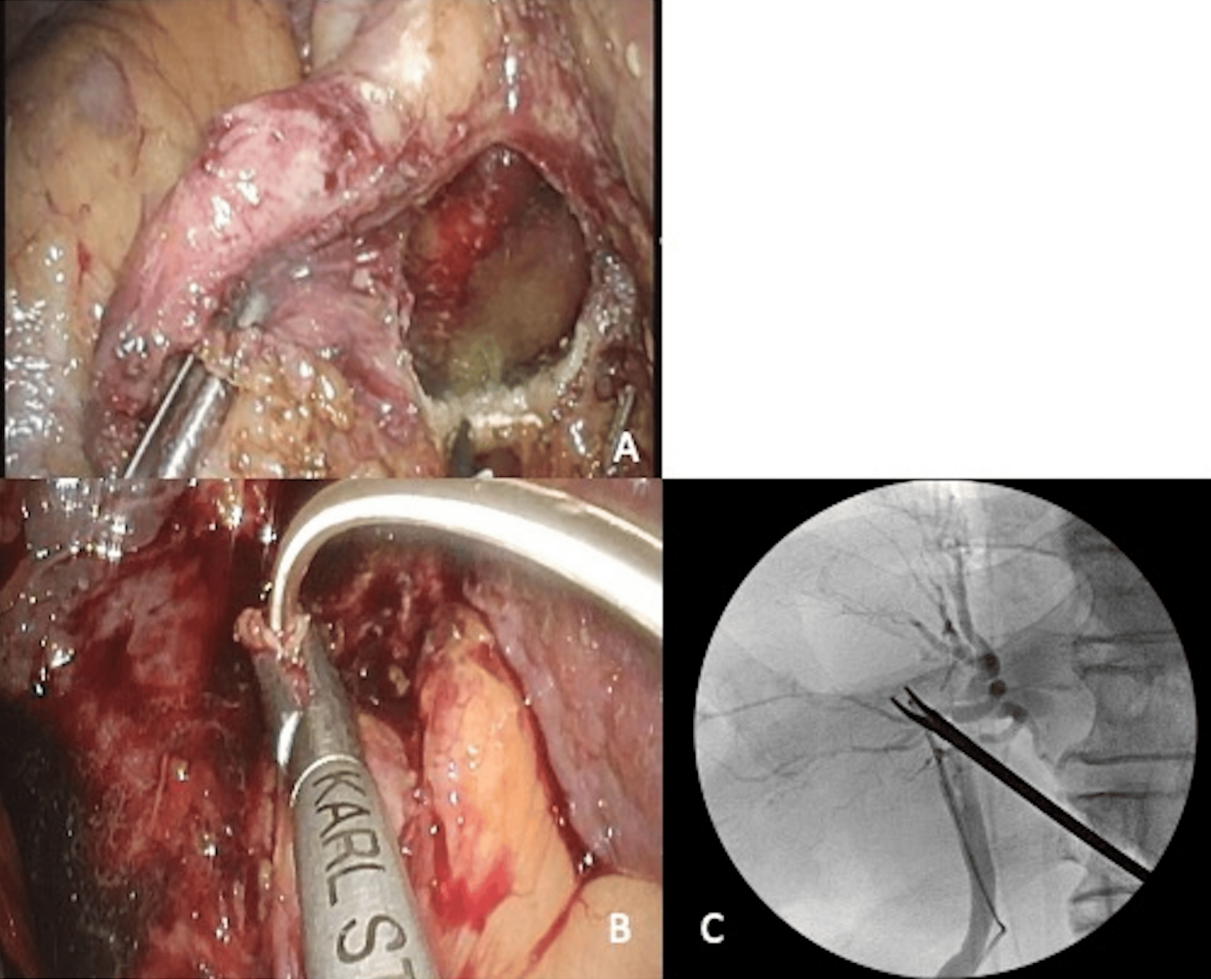
Case 2: Surgical findings and procedures. Surgical findings and procedures. (A) Because the gallbladder was deeply embedded within the liver, total cholecystectomy was not feasible; the gallbladder stones were removed while leaving the posterior wall attached to the gallbladder bed. (B) Cholangiography was performed via a C-tube inserted through the cystic duct. (C) Intraoperative cholangiography showed no leakage from the common bile duct (CBD).

Based on these findings, laparoscopic cholecystectomy was planned. Following the insertion of a 12-mm camera port through the umbilicus and three subcostal 5-mm ports (Figure 5), intra-abdominal inspection revealed that the transverse colon was severely adhered to the lateral and inferior aspects of the gallbladder. After the transverse colon was carefully mobilized, the gallbladder was found to be deeply embedded along the CBD. Subtotal cholecystectomy was performed by opening the gallbladder and leaving the posterior wall attached to the liver (Figure 6A). The cystic duct and artery were identified, and the “critical view of safety” of the gallbladder was achieved. During this process, the impacted stone was identified and retrieved via suction. Subsequent intraoperative cholangiography was performed through a transcystic tube (Figure 6B). There was no leakage from the CBD, and no stones were found (Figure 6C). Furthermore, a 5-mm round-type closed-suction drain was placed in the gallbladder bed to conclude the procedure. Unfortunately, the patient developed portal and mesenteric venous thrombosis on postoperative day 10, leading to fatal multi-organ failure.

## Discussion

Suprahepatic gallbladder is an extremely rare anatomical variant of ectopic gallbladder, reported to occur in approximately 0.026% to 0.7% of cases. This anomaly is characterized by superior displacement of the gallbladder along the common hepatic duct and is often associated with developmental abnormalities such as hypoplasia of the medial segment of the left hepatic lobe. Previous studies have demonstrated that ectopic gallbladders are frequently difficult to diagnose preoperatively, with a substantial proportion identified only during surgery. This highlights the importance of advanced imaging modalities, particularly contrast-enhanced CT and MRCP, for accurate anatomical assessment and surgical planning [1,2].

The clinical significance of suprahepatic gallbladder lies in the technical challenges it poses during the management of cholecystitis. Due to the abnormal anatomical relationship between the gallbladder and biliary tree, the posterior wall of the gallbladder may directly overlie the common hepatic duct or right hepatic duct. This distortion of Calot’s triangle increases the risk of bile duct injury during cholecystectomy, which remains one of the most serious complications of biliary surgery. Previous reports have emphasized that anatomical misidentification is a major cause of bile duct injury, particularly in cases with an aberrant gallbladder location [3,4].

In such difficult surgical situations, subtotal cholecystectomy has been increasingly advocated as a safe bailout procedure. Strasberg SM et al. demonstrated that subtotal cholecystectomy, particularly when the critical view of safety cannot be achieved, significantly reduces the risk of bile duct injury. Additional studies have supported its effectiveness in managing complex gallbladder cases with severe inflammation or abnormal anatomy [4,5]. In our second case, this strategy allowed safe management despite the challenging anatomy and adhesions.

While most previously reported cases of suprahepatic gallbladder have been managed surgically, our experience highlights the importance of individualized treatment strategies. In high-risk patients, particularly elderly patients or those with significant comorbidities, less invasive approaches should be considered. Endoscopic gallbladder drainage techniques, including ERGBD, have been reported as effective alternatives to surgery in acute cholecystitis. These techniques can serve as both definitive and bridging therapies, reducing the need for high-risk surgical intervention [6].

In our first case, ERCP-based management was successfully employed due to the patient’s advanced age, prior abdominal surgery, and high surgical risk. This approach achieved long-term control of cholecystitis without requiring cholecystectomy, suggesting that endoscopic management may be a viable option in selected patients with suprahepatic gallbladder. However, our experience also demonstrated a key limitation of ERGBD, namely the technical difficulty of cannulating the cystic duct due to acute angulation and anatomical variation. This limitation has been noted in previous reports and should be carefully considered when selecting the optimal therapeutic strategy [6].

Furthermore, the coexistence of Chilaiditi syndrome, as observed in our second case, adds an additional layer of complexity. The interposition of the colon between the liver and diaphragm can obscure the operative field and necessitate extensive adhesiolysis and careful retraction. Previous studies have reported that this condition increases operative difficulty and requires meticulous intraoperative planning [2].

Taken together, our two cases illustrate the wide spectrum of management strategies required for suprahepatic gallbladder, ranging from minimally invasive endoscopic intervention to technically demanding surgical procedures with bailout strategies. Compared with previous reports that predominantly emphasize surgical management, our findings suggest that a stepwise and less invasive approach should be prioritized, particularly in high-risk patients. Preoperative imaging, careful patient selection, and flexible decision-making are essential to optimize outcomes in this rare but challenging condition.

However, this study has several limitations, including the small number of cases and its retrospective nature. Given the rarity of suprahepatic gallbladder, further accumulation of cases is necessary to establish standardized management strategies.

## Conclusions

Suprahepatic gallbladder is an extremely rare anatomical variant that poses significant challenges in the management of cholecystitis. By prioritizing conservative treatment and endoscopic drainage where appropriate, we treated two cases: one with ERGBD stent placement and the other with laparoscopic subtotal cholecystectomy to avoid catastrophic biliary complications.
